# Predictive language processing: integrating comprehension and production, and what atypical populations can tell us

**DOI:** 10.3389/fpsyg.2024.1369177

**Published:** 2024-05-21

**Authors:** Simone Gastaldon, Noemi Bonfiglio, Francesco Vespignani, Francesca Peressotti

**Affiliations:** ^1^Dipartimento di Psicologia dello Sviluppo e della Socializzazione, University of Padua, Padua, Italy; ^2^Padova Neuroscience Center, University of Padua, Padua, Italy; ^3^BCBL–Basque Center on Cognition, Brain and Language, Donostia-San Sebastián, Spain; ^4^Centro Interdipartimentale di Ricerca “I-APPROVE–International Auditory Processing Project in Venice”, University of Padua, Padua, Italy

**Keywords:** language prediction, language production, prediction-by-production, speech-motor impairment, auditory impairment, atypical language processing

## Abstract

Predictive processing, a crucial aspect of human cognition, is also relevant for language comprehension. In everyday situations, we exploit various sources of information to anticipate and therefore facilitate processing of upcoming linguistic input. In the literature, there are a variety of models that aim at accounting for such ability. One group of models propose a strict relationship between prediction and language production mechanisms. In this review, we first introduce very briefly the concept of predictive processing during language comprehension. Secondly, we focus on models that attribute a prominent role to language production and sensorimotor processing in language prediction (“prediction-by-production” models). Contextually, we provide a summary of studies that investigated the role of speech production and auditory perception on language comprehension/prediction tasks in healthy, typical participants. Then, we provide an overview of the limited existing literature on specific atypical/clinical populations that may represent suitable testing ground for such models–i.e., populations with impaired speech production and auditory perception mechanisms. Ultimately, we suggest a more widely and in-depth testing of prediction-by-production accounts, and the involvement of atypical populations both for model testing and as targets for possible novel speech/language treatment approaches.

## 1 Introduction

Despite the seemingly ubiquitous unpredictability of language in everyday situations, research shows that there is enough regularity in language that we can anticipate (i.e., pre-activate) upcoming linguistic information at various scales, independently of its modality–spoken, written, or signed–*before* it is available in the environment for processing (Federmeier, 2007, 2021; Van Petten and Luka, 2012; Pickering and Garrod, 2013; Huettig and Mani, 2016; Kuperberg and Jaeger, 2016; Pickering and Gambi, 2018; Huettig et al., 2022; Radošević et al., 2022; Ryskin and Nieuwland, 2023). Imagine you’re at a birthday party, someone is bringing the birthday cake at the table, and you hear them saying “Let’s-”; your brain is likely to fill in the sentence with “eat the cake” before the speaker even says it. Linguistic and non-linguistic contextual cues, together with our language- and world-knowledge, guide us in anticipating what is most likely to come next. The information we can predict during processing can range from higher (conceptual) to lower (word orthography, phonology) levels and everything in between (lexical semantics, syntactic features, morphological markings) (but see Ryskin and Nieuwland, 2023, for debates on the nature of predicted representations; in particular for phonological representations, see DeLong et al., 2017; Ito et al., 2017a,b; Nieuwland et al., 2018; Nicenboim et al., 2020; Urbach et al., 2020). A variety of behavioral and neural measures have been associated with predictive language processing. Eye-tracking studies during reading revealed that words that can be predicted are skipped entirely or are fixated for a shorter interval relative to words that cannot be predicted (for a review, see Staub, 2015; for a parafoveal parallel processing interpretation of predictable words skipping, see Staub and Goddard, 2019). In visual world paradigms, where participants listen to sentences while looking at a set of pictures, the eyes fixate the target picture before the referent is mentioned in the sentence in the case of predictable words (for a seminal work pioneering this research, see Altmann and Kamide, 1999; for a review, see Huettig et al., 2011). Electrophysiological measures revealed that words embedded into sentences whose content make such words predictable elicit a different event-related potential (ERP) responses compared to words that are not predictable (i.e., a reduced N400; for reviews, see Kutas and Federmeier, 2011; Van Petten and Luka, 2012; Nieuwland et al., 2020). Crucially, time-frequency analyses of magneto/electro-encephalographic (M/EEG) data also showed differences *before* predictable words are read or listened (i.e., a power decrease in the alpha—8–10 Hz—and beta—15–20 Hz—frequency bands), more directly reflecting the prediction stage and specifically the pre-activation of upcoming linguistic information (see e.g., Rommers et al., 2017; Molinaro and Monsalve, 2018; Wang et al., 2018; Gastaldon et al., 2020; León-Cabrera et al., 2022; but see Huizeling et al., 2023 for challenges to such benchmark correlates when testing in a virtual environment). The fact that comprehenders, regardless of modality (e.g., spoken, written, signed) anticipate information yet to be available in the environment is undisputed; however, there is no consensus on the mechanisms and resources employed to this aim (e.g., Huettig, 2015; Kuperberg and Jaeger, 2016; Huettig et al., 2022; Ryskin and Nieuwland, 2023). In this manuscript we aim at unifying two points of view that we believe can provide fruitful advancement in the study of predictive processing in the language domain and that in our opinion have not been sufficiently explored.

Firstly, we focus on a group of models that aim at accounting for predictive processing during language comprehension by adopting an integrated framework in which language comprehension and production are not seen as separate domains. Such models–which for sake of simplicity we will collectively call “prediction-by-production models”, despite some architectural differences–do not propose that prediction is carried out *always and solely* by engaging resources usually devoted to language production; rather, they suggest that the language production infrastructure *can* be involved for more efficient predictions when the situation and the resources allow for it. Note that not all the models we describe here put language production as the *main* mechanism behind prediction, but they all *incorporate* it.

Secondly, we discuss how the study of sensorimotor and language systems that are dysfunctional or that are atypically developed may be particularly enlightening. Atypical populations with speech-motor and auditory perception impairments offer a fertile ground for clarifying to what extent the (spoken) language system is integrated, and consequently to determine the impact that specific deficits have on linguistic prediction. In conclusion, we bring it all together and suggest how research in predictive language processing may advance by leveraging on these two neglected aspects.

## 2 An integrated view: the role of speech production and sensorimotor processing in prediction during comprehension

Linguistic prediction is often considered a mechanism deployed during the process of understanding linguistic input, therefore at the service of comprehension. However, in the last decade, researchers have been moving away from the traditional separation between language comprehension and production as distinct domains toward a more integrative view. In this perspective, comprehension and production share representations and processes, at least partially (e.g., AbdulSabur et al., 2014; Dell and Chang, 2014; Pickering and Garrod, 2014; Silbert et al., 2014; Gambi and Pickering, 2017; McQueen and Meyer, 2019; Walenski et al., 2019; Fairs et al., 2021). The idea is not entirely new: in the past, models in which production processes are hypothesized to be implemented for speech perception were proposed, such as the Motor Theory of Speech Perception (Liberman, 1957) and the Analysis-by-Synthesis theory (Halle and Stevens, 1962). In this perspective, perceiving and categorizing speech sounds involve not only perceptual auditory mechanisms/brain networks, but also mechanisms/brain networks devoted to the production of such sounds. While the Motor Theory of Speech Perception has faced significant criticism (see e.g., Galantucci et al., 2006; Massaro and Chen, 2008), the Analysis-by-Synthesis (AxS) framework seems to have regained relevance in recent years (e.g., Poeppel et al., 2008; Poeppel and Monahan, 2011; Skipper et al., 2017; Preisig et al., 2022). AxS envisages top-down contextual inferential processes deployed by the production system that influence the bottom-up perceptual categorization of speech sounds. This view is closely related to the idea of forward models implemented for self-monitoring during speech production. During the act of speaking, the activation of representations flows in a top-down fashion through the linguistic hierarchy. A pre-verbal message is encoded, followed by the selection of lexical items to appropriately encode a linguistic message. Morphosyntactic information is then retrieved, phonological sequences are encoded and transformed into speech-motor sequences for articulation. From the motor sequences, sensory predictions are computed to monitor the speaker’s own output and correct possible errors (Indefrey and Levelt, 2004; Hickok et al., 2011; Indefrey, 2011; Tourville and Guenther, 2011; Hickok, 2012; Strijkers and Costa, 2016). In the rest of the manuscript, when mentioning “production system” (or simply “production”), we refer to the neural, cognitive, motor, and linguistic resources and mechanisms recruited and deployed to implement speech production. Therefore, we do not refer to an encapsulated system, but to a pool of resources and processes which can be partially shared to implement other tasks (McQueen and Meyer, 2019).

This top-down information flow (from higher to lower level representations) has also been proposed by some researchers to underlie predictions during comprehension of other people’s speech as well (Pickering and Garrod, 2007, 2013). There are other proposals that ascribe a prominent role to production in speech prediction (Huettig, 2015; Pickering and Gambi, 2018); however, these models are not entirely in agreement regarding which processes and representations are involved. In this section, we present three main models of “prediction-by-production,” highlighting points of convergence and divergence between them. We then provide an overview of studies on typical and neurologically healthy participants testing the relationship between speech production and prediction. Importantly, some studies suggest that motor contributions to prediction may be more crucial in challenging listening situations (e.g., Schomers and Pulvermüller, 2016; Skipper et al., 2017); therefore, we also present an overview of studies investigating the contextual top-down effects of prediction when the input is degraded and more difficult to process (e.g., speech-in-noise, vocoded speech).

### 2.1 Prediction-by-production models

The first attempts at providing an integrated view in which production processes are involved in prediction may be ascribed to Federmeier (2007) PARLO framework, and Pickering and Garrod (2007) emulator proposal. However, more extensively fleshed-out models emerged in the literature only later. Here we present three models that have been extensively developed allowing for the formulation of specific testable hypotheses (for other proposals of links between production and prediction, see also Dell and Chang, 2014; McCauley and Christiansen, 2019).

Pickering and Garrod (2013) (P&G2013) propose an integrated framework that envisions language production and comprehension as forms of action and action perception, respectively. In an attempt to overcome traditional separations, they suggest distinguishing comprehension and production based on the direction of information mapping. Specifically, production processes map from higher to lower levels of the linguistic hierarchy (e.g., from semantics to phonology), while comprehension processes map from lower to higher representational levels (e.g., from phonology to semantics). Given the top-down nature of predictions, they are therefore considered instances of production. The authors draw directly from the literature on action control, where performing an action involves predicting its outcome (Wolpert, 1997; Wolpert and Flanagan, 2001). When performing an action (e.g., moving a hand), an action command is formulated, and two parallel processes are initiated: (1) the action command is sent to the action implementer in order to perform the plan and construct the percept of the action; (2) an efference copy is generated from the action command and used to generate a forward model, predicting the sensory consequences of the action sequence (predicted percept). Percept and predicted percept are then compared, and any discrepancies are exploited to modify the action plan. As mentioned above, forward models are suggested to underlie self-monitoring during speech production (Hickok et al., 2011; Tourville and Guenther, 2011; Hickok, 2012; Franken et al., 2018; Okada et al., 2018). However, the debate on how self-monitoring is implemented is still open, often with points of view focusing on psycholinguistic (e.g., internal and external loops) or motor aspects (see e.g., Nozari and Novick, 2017; Lind and Hartsuiker, 2020; Nozari, 2020; Roelofs, 2020a,b; but see Hickok, 2012, 2014 for attempts to reconcile the psycho-neurolinguistic and the motor perspectives). P&G2013 capitalized on the parallelism between language production as a form of action and language comprehension as a form of action perception for elaborating their proposal. Forward models are generated from a production command representing the situation model and the communicative intent. Crucially, these models are extended at all levels of linguistic representations (i.e., semantics, syntax and phonology), and constitute “impoverished” [sic] representations, enabling rapid generation of predictions. This means that a person can predict the identity of the phonemes of a word but not necessarily encode their order in the sequence or predict the syntactic category of a word but not necessarily other features (e.g., grammatical gender for a noun, or tense for a verb). The key aspect in this proposal is that forward models are not employed solely to predict the speaker’s own utterances, but also to predict others’ utterances during comprehension (prediction-by-simulation). Specifically, the comprehender covertly imitates the speaker’s communicative intent and runs the intention through their own production system to generate forward models and predict the speaker’s utterance. While this is proposed as the main mechanism for prediction, the framework assumes that comprehenders can predict also through mere association (prediction-by-association), i.e., semantic priming, based on the experience in comprehending other’s sentences. In this case, the production system is not involved.

The model proposed by Huettig (2015) differs from P&G2013 in some respects. In Huettig’s (2015) proposal, predictions result from multiple mechanisms that can interact during comprehension (production-, association-, combinatorial-, simulation-based prediction; hence, PACS). Comprehenders sometimes engage their production system to predict what the speaker is likely to say, but they utilize fully-fledged production representations rather than impoverished forward models as in P&G2013 proposal. Additionally, associative mechanisms (priming) at different levels of information (e.g., semantic, phonological, and orthographic, but also at non-linguistic levels) allow for quick pre-activation of information about an incoming input. Combinatorial rules that are likely shared by comprehension and production and that are sensitive to multiple linguistic constraints further guide the pre-activation of information (e.g., determine the syntactic features of upcoming words). Finally, event simulation is exploited as heuristic strategy: the ability to imagine events and simulate their outcome based on previous experience contributes to formulate predictions. Importantly, these mechanisms represent multiple ways to predict and engaging all of them is not a necessary prerequisite for predicting. They are not encapsulated mechanisms, but they greatly interact with each other. For example, the quick pre-activation through association can serve as input for the combinatorial mechanisms responsible for higher-level structure building, which in turn can generate input for the production system. Furthermore, pre-activation from different mechanisms could sometimes be conflicting. The situational context in which comprehension takes place is essential for appropriate prediction and determines the extent to which each mechanism is exploited. According to Huettig (2015), prediction, while an important aspect, is not fundamental in language comprehension (see also Huettig and Mani, 2016), and the author stresses the need to investigate more thoroughly the mediating factors that contribute to predictive behavior (e.g., working memory resources, age, literacy).

Pickering and Gambi (2018) (P&G2018) proposed a model that is somewhere in between P&G2013 and PACS model. They suggest two routes for predicting: prediction-by-association (PA) and prediction-by-production (PP). PA automatically triggers during the incremental construction of comprehension representations and is based on the automatic spreading of activation between linguistic levels (e.g., semantic and phonological priming). While not demanding many resources, PA is somewhat inefficient as it activates all connected representations regardless of their relevance in the specific context. This is where PP comes into play. The comprehension representations computed up to that point, including those pre-activated by PA, are transformed into production representations through covert imitation. In other words, the utterance is converted into the message that the comprehender would have formulated, leading to the inference of communicative intention. This process takes into account the non-linguistic context and the comprehender’s knowledge about the speaker. The intention is then fed to the production implementer, corresponding to the stages of language production (retrieval of lexical items, lemmas, and phonological information). PP is assumed to be optional and to requires more resources than PA. Under conditions of low cognitive resources or time constraints, comprehenders may not engage the production system or go through all the stages of the production implementer, as it is time and resource consuming. This implies that slower input presentations are associated with greater contextual facilitation, which might reflect a more precise representation of the predicted word. In the framework proposed by Pickering and Gambi (2018), this association is explained by the time required to the comprehender to reproduce the sentence in their production system: when the rate of word presentation is faster, early stages of production (involving conceptual formulation and lexical selection) are more likely to be completed than later stages (such as phonological encoding). In such situations the comprehender does not have enough time to predict the form of the word (its phonology or orthography) before encountering it, although they are able to predict its semantics. The role of time constraints on “prediction-by-production” also implies that slower producers are less likely to predict words at later stages of representation or to engage in active prediction at all. P&G2018 also note that the goal of the comprehender is an additional factor influencing the likelihood of prediction, as shown by experiments manipulating task instructions (Kuperberg and Jaeger, 2016). In everyday situations, the goal of comprehension is usually to infer the message conveyed by the speaker, and in such contexts both high cognitive load and individual characteristics make active prediction less likely.

In summary, all three proposals assume an important role of priming (or prediction-by-association) as a separate and automatic route for prediction. Associative links between representations, consolidated over time, quickly pre-activate connected information at multiple levels. This activation can then be further constrained. Event simulation – and a specific form of simulation, i.e., covert imitation, – is another central aspect shared by all three proposals. However, there are differences in how simulation is viewed in each model. For P&G2013 and P&G2018 simulation is an integral part of the act of production itself and can be likened to the conceptual formulation of traditional psycholinguistic models of word production (Indefrey and Levelt, 2004; Indefrey, 2011). On the other hand, the PACS model sees simulation as a separate mechanism that optionally interacts with production representations and other mechanisms. Another distinction lies in the production mechanisms involved. P&G2013 assigns a prominent role to impoverished representations in the form of forward models, which are generated from a conceptual stage (“production command”) encoding communicative intention and situation model. In contrast, both PACS and P&G2018 propose the involvement of the fully-fledged production representations (“production implementer”). Importantly, all three models propose that the production route for prediction is optional due to its cognitive cost, even though it leads to more efficient and contextually appropriate predictions.

### 2.2 Evidence on the relationship between speech production and prediction

The increasing interest in prediction-by-production models has been accompanied by a growing literature highlighting the close relationship between production and predictive processes. Several studies have reported indirect and correlational evidence of such a relationship. For example, production abilities have been shown to partly explain individual differences in prediction: adults who are faster in categorical fluency are more likely to show patterns of neural activity associated with prediction during comprehension (Federmeier et al., 2010), while children with wider production vocabulary show higher proportion of predictive gaze shifts while listening to sentences (Mani and Huettig, 2012). This latter finding is in line with a study showing that infants are better at identifying syllables that are part of their babbling repertoire (Vilain et al., 2019), highlighting the importance of individual production capabilities in consolidating speech perception and, likely, prediction. Furthermore, from a physiological point of view, tongue evoked motor potentials have been found to be modulated by phoneme expectation during word listening (D’Ausilio et al., 2011), ERPs in a phonological priming study highlighted the involvement of speech motor regions in generating phonology-specific predictive negative slow waves (Grisoni and Pulvermüller, 2022), and neural oscillatory modulations in the alpha-beta ranges in prediction during speech comprehension show spatiotemporal correlations with modulations during speech planning (Gastaldon et al., 2020). This body of evidence suggests a contribution of speech-motor processes in prediction. Indirect evidence comes also from the so-called production effect in memory. Rommers et al. (2020) showed that the memory disadvantage for words read silently relative to words read aloud is reduced for predictable relative to unpredictable words. This suggests that in predictable contexts silent reading becomes more similar to aloud reading, thus producing similar facilitatory effects in later memory. Additionally, Hadley et al. (2020) showed that listeners are more accurate to predict turn ends when they listen to recordings of themselves or of a speaker rated similar to themselves relative to a speaker rated as dissimilar, who might be more difficult to imitate in their own production system. However, all these studies did not manipulate speech production in prediction during comprehension.

More direct evidence of prediction-by-production mechanisms comes from studies manipulating the engagement of the production system and measuring the related effect on prediction during comprehension. The study by Martin et al. (2018) studied ERPs in EEG data in participants reading sentences in Spanish while taxing the production system through articulatory suppression. The experiment manipulation capitalized on the effect by which predictable words elicit a less negative N400 (a negative deflection of the brain potentials peaking around 400 ms after word onset and emerging in centro-parietal sensors). Participants in the critical experimental group had to produce syllables while reading sentence contexts; in the control groups they either had to just move the tongue or to listen to the syllables. Critically, expected and unexpected target words differed in gender and the relative gender-specific article. The ERP results showed that the N400 effect at pre-noun articles was not significant in the syllable production group, while it was significant in the two control groups. In contrast, the N400 effect at the target noun did not vary across groups. Consistently with the importance placed on covert imitation by the prediction-by-production approach, this pattern demonstrates that production mechanisms are exploited to build lexical predictions, particularly during context reading. The early articulatory suppression and the subsequent lack of covert imitation *online*, while the sentence was unfolding over time, might have prevented participants from inferring the intended message, which in turn would have been necessary to build a forward model (Pickering and Garrod, 2013) or to pass such intention through the production system for prediction (Pickering and Gambi, 2018).

While the presence of the control groups in Martin et al. (2018) checked for the role of motor action and feedback perception, it is still possible that syllable production might have interfered with verbal working memory specifically in the experimental group. In this case, lack of prediction would be explained by the decreased availability of verbal working memory rather than by the unavailability of the production system itself. Given this potential confound, Lelonkiewicz et al. (2021) tested the role of production in prediction by exploiting a complementary approach, i.e., examining the effects of *enhancing* rather than suppressing the engagement of the production system during comprehension. In this study, participants were instructed to read high- or low constraining sentence contexts either aloud or silently, thus engaging the production system to different extents. Tasks at target word differed across experiments: word recognition, word naming, picture naming. Results showed that the facilitation given by high constraining contexts relative to low constraining ones was more prominent in the reading aloud condition, suggesting that enhancing the engagement of the production system during context reading enhances predictive processes. This result can be easily interpreted in the light of the integrated framework proposed by P&G2013. Reading aloud requires performing the action of producing the words through the articulatory system, and this action is accompanied by forward models used for self-monitoring. The availability of forward models might in turn make it easier to predict the possible continuation of the sentence, relative to a condition in which forward models are not explicitly required to perform the task. The results are also consistent with the PACS models and P&G2018, which both propose the involvement of the fully-fledged production representations. The overt articulation required by the reading aloud condition might have rendered such representations easily accessible for prediction-by-production. The study by Lelonkiewicz et al. (2021) also shed light on the nature of such predictions. Reading the sentence aloud was associated with shorter response times but did not increase accuracy, suggesting that production leads to stronger but not necessarily more accurate predictions. This pattern aligns with a prediction-by-production mechanism: when using representations from our own production system to infer the underlying message of the sentence, predictions may not always perfectly reflect the actual continuation intended by the interlocutor. Nevertheless, the production mechanism still helps to constrain the number of predicted continuations (relative to other mechanisms like association), resulting in smoother response selection and shorter response times. In the study, the strength of the interaction between predictability and reading mode (silent vs. aloud) was different according to the type of task performed on the target word: advantage for reading aloud was present in the word and picture naming tasks (at the end of a sentence frame) but absent in the lexical decision task. This pattern is consistent with the proposal that production is not the only possible mechanism for prediction, but its engagement is modulated flexibly according to the situation at hand. Specifically, when production is explicitly required for the task to perform, forward models and production representations might be more readily available to exploit prediction-by-production. In contrast, when the task is limited to comprehension (e.g., lexical decision), other forms of prediction might be implemented, possibly quicker but representationally less detailed. However, in an omnibus analysis the authors find an interaction between predictability and reading mode, but no interaction with the task/experiment. The authors therefore suggest that there is no strong evidence for differential facilitation depending on the degree to which the production system is engaged. In line with the former interpretation, Hintz et al. (2016) found that the facilitatory effects of constraining contexts on reading times are enhanced when reading trials are interleaved with picture naming trials, relative to a condition in which the two tasks are performed in separate blocks. This result suggests that prediction is favored in situations in which the production system is overall more activated. Importantly, the authors note that everyday conversations are constituted by a similar alternation of comprehension and production. Thus, prediction-by-production mechanisms might be more prominent in everyday life than in experimental settings testing solely comprehension.

It is important to note that at least one study claimed to have found evidence *against* the involvement of language production in prediction. Brothers et al. (2023) studied ERPs elicited when reading sentences that could constraint toward two possible candidates (expected and second-best). The main finding of interest here is that there was no difference in the ERPs of these two possible sentence continuations. From this empirical finding, the authors conclude that language prediction works in a different manner than language production, because, in the latter, lexical selection happens by competition via lateral inhibition (i.e., lexical items inhibit each other; Levelt et al., 1999). If language prediction worked as language production (by means of competitive pre-activation), a cost should have emerged in processing the second-best candidate, a cost that does not emerge in the data. While valuable, these findings do not automatically and incontrovertibly prove that language production has no role in prediction, because this interpretation does not automatically follow from the findings, but depends on the kind of models one assumes for production (lexical competition by lateral inhibition vs. response selection) (Spalek et al., 2013). In a model where there is no competition by lateral inhibition in word production (Dell, 1986; Mahon et al., 2007; Navarrete et al., 2014), the findings by Brothers et al. (2023) would not be taken as evidence against the deployment of production processes during prediction. Furthermore, in the study, no measure of individual language production abilities is put in relation with the ERPs measured during comprehension. Therefore, we believe the interpretation put forward by Brothers et al. (2023) should be taken more cautiously.

Overall, there is both direct and indirect evidence that production processes play a *contributory* role in linguistic prediction. The extent and relevance of such contributions need to be further specified.

### 2.3 Evidence on the role of prediction in degraded auditory input

Top-down and bottom-up information dynamically interact during speech comprehension, and several studies in the field of prediction have explored these interactions. Here, we will specifically focus on studies examining prediction in conditions where auditory perception is suboptimal. While our consideration is restricted to studies using auditory stimuli, it is essential to note that in everyday life, listeners take into account multiple sources of information, therefore language comprehension is highly visually-situated and context-dependent (Knoeferle, 2019). These factors have been shown to be especially important when the auditory input is suboptimal, due to environmental conditions or hearing difficulties (Peelle and Sommers, 2015; Beauchamp, 2016; Stevenson et al., 2017). In such situations, visual information may modulate the listener’s reliance on top-down knowledge, an observation that has not been observed in clear speech situations with ERP measures (Hernández-Gutiérrez et al., 2018). However, measures of cortical tracking of speech showed that audiovisual models better explain brain signals of participants listening to audiovisual narratives relative of audio only or visual only (Crosse et al., 2015).

Top-down processes have been proposed to aid speech comprehension in challenging listening conditions by directly influencing low-level perceptual processing, rather than being used for a post-hoc revision of the perceived word (Davis and Johnsrude, 2007). In line with this view, when listeners know in advance the content of the sentence, they perceive noisy speech as clearer and estimate the background noise as being quieter, even though this estimation should be based on basic sound perception. Additional evidence of top-down influences on perception comes from perceptual learning phenomena, i.e., the improvement in recognition of degraded speech with exposure to it. Perceptual learning generalizes to words that were not previously heard in degraded form, suggesting that it consists in “re-tuning” phonetic representations, regardless of the specific words in which they are embedded. Importantly, for words embedded in sentences (as opposed to pseudowords), listeners seem to correctly re-tune phonetic representations from the degraded input itself, with no necessity of a concomitant presentation in a clear form. The authors hypothesize that prediction might have a crucial role in this process: by pre-activating relevant representations, prediction could constrain the interpretation of degraded speech, and make the correct phonetic representation available to support perceptual retuning.

Corps and Rabagliati (2020) explored the influence of prediction on recognition of noise-vocoded (NV) speech, differentiating between phonological and semantic predictions. They presented constraining or unconstraining questions in clear form (e.g., *What colors are pandas?* vs. *What colors should I paint the wall?*) before each NV stimulus. In the first case, participants could anticipate a precise answer, including its phonological form (e.g., *black and white*); in the second case they could just anticipate the semantic field of the answer (e.g., colors). Answers were presented in NV form at 50% intelligibility and could be predictable or unpredictable with respect to the questions. Results showed that, relative to the unpredictable condition, form specific predictions did not enhance recognition of NV speech beyond predictions about the semantic field. Furthermore, both types of predictions equally enhanced perceptual learning and generalization to unseen NV stimuli. These results indicate that prediction of high-level information, such as semantics, aids speech perception and perceptual learning. Such facilitation might be especially relevant in everyday life, when listeners lack access to clear phonological forms in the presence of noisy or degraded speech, relative to semantic information from the context, which is more easily available.

The modalities in which comprehenders process the sentence context strongly influence the generation of predictions. This holds true in the auditory domain. Studies utilizing degraded sentences (as opposed to contexts presented in clear or written form, e.g., Corps and Rabagliati, 2020; Van Os et al., 2022) revealed that predictability effects interact with the level of spectral degradation. Facilitation in speech recognition is observed only for moderately degraded stimuli (e.g., Obleser and Kotz, 2010; Bhandari et al., 2021). When sentences are minimally degraded and easily intelligible, prediction has no facilitatory effects on recognition since listeners can comprehend each word regardless of its predictability (Bhandari et al., 2021). This condition can be equated to clear speech conditions, where prediction has facilitatory effects at subsequent stages of processing, increasing speed and efficiency. On the opposite end of the spectrum, high levels of degradation make sentence context hardly intelligible, hindering listeners’ ability to use the context to form predictions. In this case, even when listeners can understand the context, the increased listening effort required to process the auditory input leaves few cognitive resources available for prediction (Bhandari et al., 2021). This view is in accordance with prediction-by-production accounts, which consider production as a costly and optional mechanism for prediction, dependent on the features of the bottom-up input. Furthermore, at moderate levels of intelligibility (i.e., with recognition scores significantly above chance but below ceiling), there is evidence that excitability in the speech-motor areas is higher than when speech is fully intelligible, and such excitability is related to identification accuracy of distorted syllables (Nuttall et al., 2016). These results suggest that motor activation plays a critical role in the comprehension of distorted speech, supporting the notion that prediction can aid speech perception through production mechanisms.

The interaction between predictability and level of intelligibility has also been studied also by exploiting speech masked with noise. Marrufo-Pérez et al. (2019) tested speech recognition, manipulating the stimuli around the individual speech reception threshold (SRT) which is the level of signal-to-noise ratio at which a participant recognizes 50% of the sentences. At this intelligibility level, recognition scores were found to decrease as the sentence unfolded, with initial words recognized better than the words in following positions. This decrease in recognition scores suggests that increase in word predictability is not beneficial at SRT levels, possibly even being detrimental to speech recognition. The authors explored this possibility by modeling the probability of recognizing each word given the recognition (or not recognition) of the previous one. This analysis showed that words were significantly less likely to be recognized when the preceding word was missed or misunderstood, and that this effect overrode the beneficial effect of recognizing the preceding word. However, with increasing intelligibility above the SRT the detrimental effect gradually disappeared, indicating that when speech is clearer listeners are less likely to be misled by the preceding word and rely more on the bottom-up input they perceive (for a similar discussion see also Van Os et al., 2022). Overall, this pattern indicates that beneficial or detrimental effects of predictability strongly depend on the level (and type) of noise as well as on the individual sensitivity to speech-in-noise (indicated here by the individual SRT). In real-life situations, speech-to-noise ratios are sufficiently higher than SRTs for normal hearing listeners, but they might not be so for hearing-impaired listeners; thus, predictability might have different effects in normal and impaired hearing listeners.

It is interesting to note that also older adults exploit contextual information and stored knowledge to facilitate speech comprehension, thus highlighting the relevance of context and prediction in situations in which auditory perception has gradually become less efficient due to normal aging (Pichora-Fuller, 2008). Furthermore, a recent study on audiovisual syllable perception in noise in older adults unveiled the crucial contribution of sensorimotor regions for successful performance: participants who received musical training in their life performed better than those who did not have such training, and this facilitation was due to stronger engagement of sensorimotor regions (Zhang et al., 2023).

In conclusion, experimental studies show that prediction can aid speech perception when the auditory input is sub-optimal; however, the extent by which such facilitation is possible and efficient strongly depends on the features of the bottom-up input. Together with evidence suggesting the involvement of the speech motor system in aiding perception especially in noisy or auditory degraded conditions (for a review, see Skipper et al., 2017), we believe that a further understanding of how speech production processes contribute to prediction is to be pursued, in particular to empirically test the models presented in section “2.1 Prediction-by-production models.”

## 3 What atypical and clinical populations can tell us about prediction

In analogy with the classical approach in cognitive neuropsychology (Caramazza and Coltheart, 2006), studying how a particular process emerges in populations with impairments in different relevant mechanisms can help us understand the extent to which the impaired mechanism matters in the intact brain. For instance, studying linguistic prediction in populations with impairments in processes (and brain structures and function) mainly devoted to speech production can help us specify their contribution to prediction during comprehension. Conversely, studying prediction in populations with deficits in encoding the auditory input due to acquired or congenital dysfunctions can shed light on the role of the quality of the sensory input in relation to top-down cognitive processes and demands. In the following section, we discuss the limited literature available, specifically focusing on people with developmental stuttering and people with Parkinson’s disease for production, and cochlear implant mediated speech in deaf people for perception. Such populations provide a unique testing ground for theoretical models. Importantly, findings stemming from such research may turn out to have clinical applications for novel treatment approaches.

### 3.1 Inefficient speech production

One population that can provide valuable insights on the role of the speech-motor system to prediction during comprehension is people who stutter. Developmental stuttering (DS) is a multifactorial neurodevelopmental disorder that disrupts speech fluency, leading to blocks, repetitions, and prolongations of sounds (Smith and Weber, 2017). The neuroimaging literature consistently showed that DS is associated with atypical gray and white matter patterns in regions involved in speech-motor control (for a review, see Etchell et al., 2018). Specifically, people with DS exhibit an inefficient mapping between motor and sensory predictions, an overly inhibited feedforward motor system, and an inefficient internal timing/sequence organization (e.g., Alm, 2004; Max et al., 2004; Civier et al., 2013; Busan, 2020). Such dysfunctions emerge as atypical modulations of alpha-beta activity in the electro-magnetoencephalographic signal during speech production (e.g., Mersov et al., 2016; Mock et al., 2016; Jenson et al., 2018). Therefore, these processes and their electrophysiological correlates are relevant from the point of view of the prediction-by-production models we discussed above. To explore whether impaired production causes altered predictive processing, Gastaldon et al. (2023) investigated prediction during spoken language comprehension while recording EEG in adults with DS. The study found that, compared to age-matched fluent speakers, adults who stutter showed atypical patterns of electrophysiological correlates of word prediction during comprehension, namely a reduced N400 effect and an increase of alpha-beta power in premotor and frontal regions. The former finding suggests that the way in which predicted and unpredicted words are integrated is different between controls and adults who stutter. The second finding implies that this difference may be due to less detailed predictions, likely as a result of a lack of (or reduced) involvement of the inefficient speech-motor network. Furthermore, correlations of alpha/beta activity between comprehension and production tasks in cortical regions of interest showed that, while positive correlations were found in left inferior frontal and temporal regions in fluent participants, suggesting possible convergence of mechanisms in the two tasks in those cortical regions (see also Gastaldon et al., 2020), no consistent correlations were found in adults with DS. This further suggests that when speech-motor processes are sub-optimal, they are not appropriately co-opted for prediction during comprehension. Speculatively, such “impoverished” predictions may emerge as lexico-semantic predictions with no phonological and sensory component.

Another clinical population that can shed light on the role of motor control in linguistic prediction is people with Parkinson’s disease (PD). PD is a neurodegenerative syndrome that initially affects motor control, leading to bradykinesia, rest tremor, and/or rigidity, but later progresses to also impact cognitive and emotional-affective domains (Bloem et al., 2021). León-Cabrera et al. (2021) studied prediction during reading in people with PD, both with normal cognition and with mild cognitive impairment, compared to matched controls. The authors recorded EEG and focused on two ERPs relevant for prediction: the prediction negative potentials (PNP) before final words and the N400 at the final word. The authors found no group differences between controls and people with PD and normal cognition. However, differences were found between people with PD with and without cognitive impairment: the latter showed no PNP effects and a prolongation of N400 effects, suggesting that general cognitive decline is responsible for impaired predictive processing. Interestingly however, the PNP effect in people with PD (including those with normal cognition) was correlated with verbal fluency. This is also in line with findings on healthy older adults, showing that participants with high verbal fluency generate a pattern of prediction-related ERPs effects similar to that of young adults, differently from older adults with low fluency scores (Federmeier et al., 2010; Dave et al., 2018). While verbal fluency encompasses a variety of functions, it is considered a measure of speech production efficiency, further linking language production with prediction during language comprehension. This suggests that further investigation into the relationship between speech production in PD and linguistic prediction may be valuable. Note that the authors did not analyze oscillatory activity, which may capture effects that are not easily detected with ERPs (Gastaldon et al., 2020). Given the relevance of alpha-beta activity also in PD (Belova et al., 2021; Johari and Behroozmand, 2021), we think this complementary approach could yield fruitful insights.

It is interesting to note that the alpha-beta aberrant activity that is observed both in DS and PD has been linked to a dysregulation of dopamine levels in the cortico-basal ganglia-thalamo-cortical loop that is responsible for initiating speech (Chang and Guenther, 2020; Alm, 2021). Studying the role of neurotransmitter levels in key speech production regions may be a promising research direction to further understand the neurophysiological bases of the processes that are hypothesized to be involved in predictive language processing.

To the best of our knowledge, the literature on such topic is still very limited and hopefully developing. Nevertheless, we argue that testing the effects of impaired speech/language production mechanisms on prediction is a first step for delineating a more complete picture of predictive processing and the language system as a whole, beyond the separation between comprehension and production.

### 3.2 Sub-optimal auditory input: cochlear implant-mediated speech

To investigate the extent to which people rely on predictive processing based on the quality of auditory input, researchers can examine deaf people with cochlear implant (CI). CIs are neural prostheses that are commonly used to treat severe and profound sensorineural deafness, enabling sound perception even in presence of damages or congenital malfunctioning of the cochlea (Macherey and Carlyon, 2014). CIs support speech perception in deaf people and play a crucial role in the development of spoken language skills in deaf children, particularly if they are implanted before the age of three (Hunter and Pisoni, 2021). However, while in many cases CIs provide sufficient information for speech comprehension, the auditory input conveyed is qualitatively degraded compared to the natural input of a healthy cochlea (for an in-depth discussion of the acoustic features of the CI input, see Hunter and Pisoni, 2021). In this section, we will review studies comparing predictive processes in CI users and normal hearing (NH) listeners, to elucidate the role of the auditory input in prediction. We will consider both studies on postlingually and prelingually deaf CI users; however, it is worth noting that speech processing mechanisms might differ between these two populations. Postlingually deaf CI users have acquired language with a normal auditory input and later adapted to the CI input after experiencing hearing loss. On the other hand, prelingually deaf CI users, who represent the majority of participants in studies with children, have developed language abilities solely on the basis of the CI input, potentially leading to differences in linguistic and cognitive development compared to NH peers (see, for example, Hunter and Pisoni, 2021). Thus, the features of the specific sample tested must not be overlooked.

In line with studies on NH listeners using degraded sentences (e.g., Obleser and Kotz, 2010; Bhandari et al., 2021), some authors propose that the limited amount of speech cues available to CI users might compromise their capacity to represent context efficiently and generate predictions about upcoming words (Baskent et al., 2016). Moreover, more effortful speech processing might result in lack of time and cognitive resources sufficient to use semantic cues and predictive processes (Holt et al., 2021; see also Bhandari et al., 2021). Winn (2016) utilized pupil dilatation as an online measure of cognitive effort during sentence listening. Postlingually deaf CI users were compared with NH controls who listened to clear speech and spectrally degraded speech matching the intelligibility of the CI input. The results indicated that constraining sentence contexts were associated with a reduction in pupil dilatation, supporting the notion that efficient prediction reduces the overall cognitive resources expense. The effect was found in both groups of participants, but with different timing: pupillary reduction appeared before the end of the sentence for NH listeners when exposed to clear speech, and after the end of the sentence for CI users and for NH listeners exposed to degraded speech. A possible explanation might be that context is not used predictively by CI users, but rather to facilitate a post-hoc restoration of misperceived words in the sentences. Highly constraining contexts allow listeners to infer words that have been misperceived during online processing, potentially leading to a release from the cognitive effort exerted to maintain multiple lexical options active.

A study conducted by Nagels et al. (2020) examined postlingually deaf CI users and NH controls in a visual word paradigm. Participants were shown images of the target word and of distractors, including phonological and semantic competitors of the target word. When predictive strategies were employed, looking times toward semantic competitors increased, while looking times toward phonological competitors decreased compared to a not-constraining condition. This pattern was observed for both the CI and the NH groups, but quantitatively different according to the group. In particular, CI users showed a higher proportion and duration of fixations toward the competitors relative to NH controls. The group of CI users also showed great individual variability in the size of the effect of constraining context. The authors further explored these individual differences by administering an auditory lexical decision task, from which they extracted an individual measure of uncertainty in the interpretation of the speech signal (i.e., “lexical uncertainty”). This measure reflects difficulties in lexical competition and in subsequent lexical access from a speech input. In the visual world paradigm, participants with higher lexical uncertainty displayed more pronounced differences between constraining and not-constraining conditions, suggesting that reliance on context might serve as a compensatory strategy particularly for the individuals who face difficulties in the interpretation of the auditory input. At the same time, higher lexical uncertainty was associated with longer time courses of lexical competition, suggesting that the delay found in the group analysis was primarily driven by this subgroup of CI users.

In addition to time and cognitive resources, a crucial factor in determining the engagement of prediction (and of costly mechanisms as prediction-by-production in particular) is its utility (Kuperberg and Jaeger, 2016). When interpretation of the speech signal is challenging, misunderstandings of previous words might lead to prediction errors (see e.g., Marrufo-Pérez et al., 2019). In such a case, delaying and reducing commitment to predictions might be an adaptive strategy to avoid the costs of revising predictions that turn out to be incorrect (Blomquist et al., 2021). Supporting this notion, a study on single-word recognition revealed that prelingually deaf adolescents with CI adopt a wait-and-see strategy, delaying access to the relevant lexical representation until the auditory input becomes sufficiently informative (McMurray et al., 2017). Importantly, the same study found that NH controls exposed to degraded input exhibited a similar pattern of behavior, suggesting that delaying lexical access might be a strategy driven by the specific features of the input. Blomquist et al. (2021) tested the presence of predictive strategies in children with and without CI in a visual world paradigm, and found that constraining contexts, compared to unconstraining contexts, elicited faster looks to the target and reduced looks to the phonological competitor before target onset in both groups. However, children with CI showed a smaller proportion of looks to the target and a greater proportion of looks to the competitor relative to controls. These results indicate that children with CI can predict, but they might reduce commitment to the formed predictions relative to their NH peers.

An important consideration when interpreting findings from prelingually deaf CI users is that early exposure to the CI input might lead to significant differences in linguistic development (Hunter and Pisoni, 2021). For example, individual vocabulary measures seem to partly explain group differences in predictive processes, suggesting that children with CI with better linguistic knowledge predict more similarly to their NH peers (Blomquist et al., 2021). Interestingly, a study by Holt et al. (2021) with the visual world paradigm did not find any group difference between NH children and CI users, who exhibited similar timing of lexical access and speech processing in both constraining and not-constraining conditions. This result indicates that there might be a great variability in individual outcomes after early implantation, both in terms of linguistic abilities and neural adaptation (Hunter and Pisoni, 2021). Indeed, for children with reduced difficulty in identifying words, building and committing to predictions might be as convenient as for their NH peers.

Another source of variability might be the specific contextual cues present in the sentence. For example, Davies et al. (2023) tested the use of syntactic cues, especially plural/singular subject-verb agreement, with a variation of the visual world paradigm in which children looked at pictures of singular and plural subjects, one of which was coherent with the sentence they heard. The results showed that children with CI use subject-verb agreement to facilitate lexical access, but they do so more slowly than their NH peers. Importantly, participants in this study greatly overlapped with the ones in Holt et al. (2021), suggesting that the difference in results is driven by the type of information predicted (i.e., semantic vs. syntactic) rather than by differences between participants. Specifically, syntactic cues might be more challenging to recognize when processing speech through a CI.

In conclusion, results from studies on CI users are consistent with studies on NH listeners using degraded speech, suggesting that the quality of the auditory input can impact listeners’ use of optional mechanisms of prediction. Results also suggest that prediction is not an all-or-nothing phenomenon: there might be a spectrum of predictive strategies implemented differently between individuals. In the case of CI users, not only the auditory input might play a role, but also its integration with information from other sensory modalities, e.g., visual cues. CI users have been shown to have better integration abilities, especially in presence of higher linguistic information (Rouger et al., 2007; Strelnikov et al., 2009; Stevenson et al., 2017). Currently ongoing research from our lab is going to explore predictive processes in CI users with less artificial stimuli, exploring possible interactions with audiovisual integration processes, and the role of the speech-motor network in aiding prediction and integration.

## 4 Discussion and future perspectives

The role of prediction in language comprehension has long been recognized and it has been a hot topic for many years now. In this review and perspective we first focused on prediction-by-production frameworks which have been developed in recent years in an attempt to reconcile the traditional and arbitrary divide between language comprehension and production research (McQueen and Meyer, 2019). One crucial aspect that emerges from the proposals summarized in section “2.1 Prediction-by-production models” is that the “production route” is costly and optional: under this proposal, production processes (ranging from intention formulation, lexical selection to phonological encoding and motor programming) are deployed to reinforce prediction only when it is convenient (i.e., when there is an advantage in doing so) and according to the specific situation at hand, in contrast to passive spreading of activation, which happens automatically and without costs. This picture envisages at least a “dual route” (or “multi route” as in Huettig’s PACS model) view of prediction: one is more automatic (spreading of activation / priming / prediction-by-association), the other is costlier (action-/simulation-based prediction / prediction-by-production). Dual-routing cognitive processes is a classical “easy” solution for cognitive scientists to explain complex nonlinear or apparently contradictory behaviors, successfully applied in different fields of cognition such as decision making (heuristic vs. analytic; Samson and Voyer, 2012) and word reading (lexical vs. phonological routes; Humphreys and Evett, 1985). It might be even more plausible to consider the presence of multiple prediction pathways, such as those proposed in the PACS model, under the assumption that various input features can be predicted using distinct mechanisms. For instance, one can easily predict that a noun will follow the English word “the” (maybe after some adjectives), or that after the word “an” a word-initial vowel will follow. In other instances, one can predict the specific phonemes in a word after the uniqueness point has been reached. Other kinds of predictions require rather complex inferential processes and specific world-knowledge (e.g., in the sentence “Caterina is planning to write a grant proposal on vocal communication in…,” one can predict the word “crows” if they know the specific Caterina it’s been talking about and her specific scientific interests). Other predictions require a more linguistically informed long-term memory information for idioms (e.g., “nine” comes after the sentence fragment “After receiving the results of the exam, Giovanni was on cloud…”). Intuitively, it appears clear that all these forms of linguistic predictions may rely on different processes and resources, linguistic and non-linguistic, but research still has not tackled this.

Given the picture above, where at least two routes are outlined, one question that arises then is how this view can be reconciled with the predominant framework that envisages the brain as a “predictive machine,” namely predictive coding (PC) (Friston, 2005, 2010; Bar, 2009; Clark, 2013). Despite being often cited in the literature in language processing, PC is often and wrongly used as a synonym for “predictive processing” in general (as pointed out in Nour Eddine et al., 2022). Instead, PC proposes very specific neural computations as a fundamental algorithm for brain functioning, according to which the brain constantly generates predictions to infer the hidden causes of sensory phenomena in the environment, by exploiting (and updating) internal generative models. The aim of the brain is to minimize prediction error (the discrepancy between top-down predicted representation and bottom-up signal). This algorithm is proposed to be instantiated in a very specific cortical hierarchy, with state units that encode predictions sending information to lower-levels, and separate error units sending information upward to update the internal model. This formulation makes prediction an automatic process that happens constantly because it is a necessity of the biological system and its core mechanism for encoding information. An increasing number of studies have highlighted that predictive processing seems to be a key element of language processing emerging beyond controlled experimental paradigms, but also in more naturalistic listening and reading situations (e.g., Donhauser and Baillet, 2020; Schrimpf et al., 2021; Brodbeck et al., 2022; Heilbron et al., 2022; Tuckute et al., 2024). Furthermore, recent computational models that strictly adhere to the PC algorithm successfully simulated ERP modulations (especially of the N400) for a variety of effects, although not all of them and with architectural limitations (e.g., some layers are directly fed with values instead of being the results of previous computations, thus failing to replicate other aspects of language processing that are exploited for prediction) (Nour Eddine et al., 2022, 2024). This body of evidence consistently supports the notion that top-down predictions are regularly generated, at least in literate and typical populations, and potentially in alignment with PC algorithms.

How do prediction-by-production models fit in this perspective? According to Pickering and Garrod (2013) and Pickering and Gambi (2018), the production route is the one that provides the best predictions in terms of appropriateness of context and specificity (i.e., down to phonological and sensory information); however, it may not be always possible to implement production-based predictions. Multiple routes—like in the PACS model—seem to be even more difficult to reconcile with a limited set of cortical operations put forward by PC. We believe this is a crucial aspect that research in psycholinguistics and cognitive neuroscience of language need to tackle, that is, to better understand (or confute) the contribution of production processes in prediction in order to also better understand the relation with other proposed mechanisms of prediction and general brain functioning.

Here is why we propose that the study of atypical populations, which is currently underdeveloped as we highlighted in Section “3 What atypical and clinical populations can tell us about prediction,” should be further pursued, since it could contribute to “complete the puzzle.” In particular, investigating prediction in the presence of inefficient language/speech production processes can shed light on what is the nature of predicted representations, possibly identifying a grading of preactivated information. For instance, while providing evidence that developmental stuttering negatively impacts prediction and tentatively providing an explanation on the basis of the neural and behavioral emergence of stuttering, the study by Gastaldon et al. (2023) leaves open the question of what the exact impact on predicted representations is. It is possible that inefficiency of forward model mapping in DS yields unspecified predictions from a sensory point of view. Further research should clarify this point. One could further envisage a research enterprise in which a spectrum of language and speech-motor deficits could be studied, ranging from central linguistic processes (impairments in lexical selection and phonological encoding, such as in certain profiles of aphasia or developmental language disorders; Denes and Pizzamiglio, 1998; Schwartz, 2010) to more peripheral deficits (such as lack of facial/oral muscle control). For instance, studies on facial paralysis seem to suggest an impairment in emotional faces processing (Sessa et al., 2022; Japee et al., 2023). As a parallelism, one could argue that paralysis of muscle districts responsible for sound production may impact sensory aspects of linguistic processing. A study on people with Moebius syndrome—a congenital neurological disorder causing complete face paralysis which causes impairments in articulating speech sounds (Pamplona et al., 2020)—did not find support for the hypothesis that lack of muscle control impacts lip reading, an aspect closer to face-to-face linguistic communication (Vannuscorps et al., 2021). This suggests that it is unlikely that damage to or atypical development of more peripheral mechanisms of speech and language production affects relevant aspects of speech processing. This remains an empirical question to explore. Conversely, further studying speech comprehension in people with varying degree of hearing loss and deaf people with cochlear implant can clarify the role of top-down preactivation. Importantly, what can be learnt from research on production deficits may be important from a clinical standpoint for populations with hearing impairments. For instance, given the inherently suboptimal nature of the acoustic input conveyed by the implant and the involvement of speech-motor simulation in difficult listening situations (Skipper et al., 2017), it may be particularly relevant for people with CI to exploit production-based mechanisms for prediction. For instance, Sherafati et al. (2022), by using functional near-infrared spectroscopy, found that the left prefrontal cortex was more activated in CI users relative to controls when listening to speech. The authors interpreted this activation as domain-general processes supporting perception and comprehension. However, given the extension of their region of interest into the inferior frontal gyrus, they cannot exclude other language-related processes. Future research should clarify whether CI exploits their production machinery to support comprehension, and under which circumstances. Overall, we believe that the study of atypical/clinical populations, despite the inherent difficulties (e.g., arduous participant recruitment, small sample sizes, etc.), can be fruitful both from a theoretical standpoint, but importantly also from a societal and applied perspective, by fostering public awareness and a deeper understanding of the speech/language deficits, and leading to new approaches to therapy.

In conclusion, we propose that the prediction-by-production perspective needs to be taken into consideration more systematically, faced in a theoretically rigorous way, and more widely empirically tested, also by including atypical populations that can help clarify the picture. This can contribute both to a better understanding of the language system and its relation with mechanisms of brain functioning, and suggest new venues for speech and language intervention.

## Author contributions

SG: Conceptualization, Project administration, Writing – original draft, Writing – review and editing. NB: Conceptualization, Writing – original draft, Writing – review and editing. FV: Conceptualization, Supervision, Writing – review and editing. FP: Conceptualization, Funding acquisition, Supervision, Writing – review and editing.
